# Beta-hydroxy-beta-methyl-butyrate blunts negative age-related changes in body composition, functionality and myofiber dimensions in rats

**DOI:** 10.1186/1550-2783-9-18

**Published:** 2012-04-18

**Authors:** Jacob M Wilson, Samuel C Grant, Sang-Rok Lee, Ihssan S Masad, Young-Min Park, Paul C Henning, Jeffery R Stout, Jeremy P Loenneke, Bahram H Arjmandi, Lynn B Panton, Jeong-Su Kim

**Affiliations:** 1 Department of Nutrition, Food and Exercise Sciences, The Florida State University, Tallahassee, FL, USA; 2 Department of Health Sciences and Human Performance, The University of Tampa, Tampa, FL, USA; 3 The National High Magnetic Field Laboratory & Department of Chemical & Biomedical Engineering, The Florida State University, Tallahassee, FL, USA; 4 Military Performance Division, United States Army Research, Institute of Environmental Medicine, Natick, MA, USA; 5 Biomedical Engineering Department, College of Engineering, King Faisal University, Al-Ahsa, Saudi Arabia; 6 Sport and Exercise Science, College of Education, University of Central Florida, Orlando, FL; 7 Department of Health and Exercise Science, University of Oklahoma, Norman, Oklahoma, USA

**Keywords:** Beta-hydroxy-beta-methylbutyrate, Aging, Fat-free mass, Strength, Sarcopenia

## Abstract

**Purpose:**

To determine the effects of 16 wk. of beta-hydroxy-beta-methylbutyrate (HMB) administration on age-related changes in functionality and diffusion tensor imaging (DTI) determined myofiber dimensions.

**Methods:**

Twelve young (44 wk.), 6 middle-aged (60 wk.), 10 old (86 wk.), and 5 very old (102 wk.) male Fisher-344 rat's body composition and grip strength were assessed at baseline. Following, 6 young, 6 middle-aged, 5 old and 5 very old rats were sacrificed for baseline myofiber dimensions and gene transcript factor expression in the soleus (SOL) and gastrocnemius (GAS). The remaining 6 young and 5 old rats were given HMB for 16 wk. and then sacrificed.

**Results:**

Fat mass increased in the middle-aged control condition (+49%) but not the middle-aged HMB condition. In addition, fat mass declined (-56%) in the old HMB condition but not the old control condition. Normalized strength declined and maintained respectively in the control and HMB conditions from 44 to 60 wk. and increased (+23%) (p < 0.05) from 86 to 102 wk. in only the HMB condition. Declines occurred in myofiber size in all muscles from 44 to 102 wk. in the control condition(-10 to -15%), but not HMB condition. Atrogin-1 mRNA expression in the SOL and GAS muscles was greater in the 102-wk control condition than all other conditions: SOL (+45%) and GAS (+100%). This elevation was blunted by HMB in the 102 wk. old SOL. There was a condition effect in the SOL for myogenin, which significantly increased (+40%) only in the 102-wk. HMB group relative to the 44-wk. group.

**Conclusions:**

HMB may blunt age-related losses of strength and myofiber dimensions, possibly through attenuating the rise in protein breakdown.

## Backgrounds

In the 20th century, the United States experienced a 57% increase in lifespan (from 49.2 to 76.5 years) [1]. With continued growth per annum life expectancy is projected to rise to approximately 80 and 84 years of age in women and men, respectively, by the year 2050 [1]. It has been shown that there is a 30% loss of muscle tissue that occurs from the 5th to 8th decade of life [2]. This progressive age-related loss of muscle tissue, strength, and function is termed sarcopenia [3]. Sarcopenia is associated with a greater likelihood of disability, functional impairment in activities of daily living [4,5], increased incidence of falls, insulin resistance [6], and hip fractures [7]. Each of these factors appears to contribute to a projected doubling of 65 year olds becoming limited to nursing homes by 2020 [1]. It is projected that as individuals aged 65 years or older increase from 13% to 20% of the population from 2000 to 2020, a paralleled 2 to 6 billion dollar increase in hip fracture expenditures is projected to occur [7]. Therefore, a better understanding of the factors that cause slow or possibly reverse sarcopenia is critical for improving the quality of life in elderly populations, as well as blunting the estimated increase in health care costs.

Within the last decade, long-term essential amino acid (EAA) supplementation has been demonstrated to serve as a possible treatment and/or prevention for the muscle loss associated with aging [8-13]. Leucine has been found to be a crucial component within the EAA complex to possibly attenuate the progression of muscle wasting [10,12]. One of reasons that leucine may attenuate muscle wasting comes from its conversion to beta-hydroxy-beta-methylbutyrate (HMB) [14]. However, only 5% of leucine is metabolized into HMB [15]. Thus, an individual would need to consume 60 to 120 g of leucine in order to obtain the most frequently administered dosages (3 to 6 g, respectively) for this supplement in research studies. HMB has attenuated muscle wasting in numerous clinical situations including those involving cancer [16-19], human caloric restriction [20], and limb immobilization [21]. HMB also has been found to counter age-related losses in limb circumference [9], upper and lower body strength [8], and functionality in activities of daily living [9]. Moreover HMB has been demonstrated to signal the simultaneous increase and decrease in protein synthesis and proteolysis in both aging and clinically cachexic conditions [16,22]. Given HMB's capacity to subsequently enhance and depress anabolic and catabolic pathways [16,22], HMB would be a good candidate as a dietary supplement to partially reverse deficits in net anabolism in sarcopenic muscle following RET.

To our knowledge, no research has investigated the effects of HMB on age-related changes in muscle cell (myofiber) size. Moreover, no study to date has compared and contrasted if differential responses exist between young and older individuals to HMB consumption. Therefore, the primary aim of this study was to determine the effects of 16 wk. of HMB administration in young and old rats on age-related changes in body composition, functionality, and myofiber dimensions using advanced *ex vivo *magnetic resonance (MR) imaging techniques and the potential molecular mechanisms mediating these effects.

## Methods

### Animals and overview of experiment

**Figure 1:**
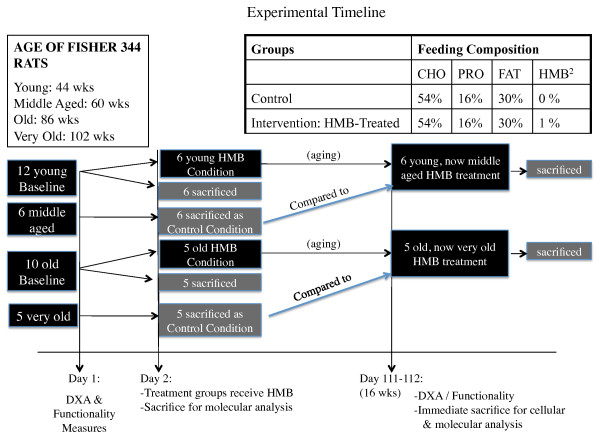
**Schematic of experimental timeline for the experiment**.

All procedures in this study were approved by our institutions Animal Care and Use Committee. Fourteen young (44 wk.), 7 middle aged (60 wk.), 14 old (86 wk.), and 7 very old (102 wk.) male Fisher 344 rats were used in the study. However, death due to the aging process as well as general anesthesia during various imaging processes resulted in a remainder of 12 young (44 wks.), 6 middle aged, which served as the control (60 wk.), 10 old (86 wk.), and 5 very old, which served as the control (102 wk.) animals that completed the study (see Figure 1 for timeline), which still met the criteria for our original sample size determination (see power analysis below). Each animal was assessed for functionality (grip strength and motor performance using incline plane) as well as lean, fat, and total body mass using dual-energy X-ray absorptiometry (DXA) pre- and post-treatment (see Figure 1 for experimental design). After baseline measures, 6 young, 6 middle aged control, 5 old, and 5 very old control rats were anesthetized and their right gastrocnemius (GAS) and soleus (SOL) muscles were isolated, blotted, and quickly frozen in liquid nitrogen for later *in vitro *molecular analysis. After isolating muscles from the right hind limb, a cardiac perfusion protocol was implemented to drain blood from the rat's body. Following, the left GAS and SOL muscles of the rats were harvested and directly immersed in 4% paraformaldehyde for an *ex vivo *analysis of myofiber dimensions. Remaining young (44 wk.) and old (86 wk.) rats were given HMB (0.46 g/kg/d) for 16 wk. After the supplementation period, the remaining rats were assessed for post-treatment measures in body composition and functionality and then sacrificed for *in vitro *molecular *and ex vivo *MR analyses.

### HMB administration

All animals were raised in our laboratory prior to experimentation, therefore giving us a strong basis for how much HMB should be added to their food. Typically, daily food consumption values ranged from 15-25 g/day for a 250 g rat (or 60-100 g feed/kg BW). Based on their average diet, the HMB dosage was calculated as ~1% CaHMB (Metabolic Technologies Inc., Ames, Iowa, USA), to achieve an ~0.50 g HMB/kg BW/daily dose [20]. Based on previous human studies, and assuming a rodents metabolism are at least 6 times more than humans, we chose a 6 gram metabolic equivalent HMB intervention (the upper limit given to humans in research [23]) and calculated a human-to-rodent conversion to provide an appropriate, and safe dosage for each animal [20]. Daily food consumption of rats was measured every 6th day by weighing the food remaining and subtracting it from the amount that was administered. Upon termination of this study, the average kilocalories (kcals) for total food consumed, as well as for each macronutrient, were calculated.

### Body composition

Dual-energy X-ray absorptiometry (DXA) was performed using a Lunar QDR system (iDXA, Lunar Corp., Madison, Wisconsin, USA) with specific software (version V8-19a) and an internal standard adapted for small animal scans. Total body mass (TBM), lean body mass (LBM), and fat mass (FM) were measured on all animals' pre and post 16 wk. of HMB administration.

### Functionality measures

The grip strength test was used as a measure of limb strength [24]. In this procedure, the rats were positioned in front of a force gauge (DFS-101 Force gauge, AMETEK TCI, CA, USA) so that they could grasp the tension sensitive steel bar of the device with their forelimbs. After visual observation of gripping, the researcher gently pulled back on the rat's tail until it released its hold on the bar. Force produced was measured in grams. Three trials were performed by the same experienced investigator for each rat throughout the study for consistency and the greatest force was recorded as maximum grip strength, which was then normalized to body mass of each rat.

The inclined plane test was used to assess sensory motor function and hind limb strength [25]. Performance was determined as the rats' ability to maintain their body position for 5 sec on an inclined plane, while the angle of the surface was changed from 20° to 60° at 2° intervals, with a rest period of at least 5 min.

### Muscle isolation

Both right and left hind limb muscles were collected in the National High Magnetic Field Laboratory (NHMFL): one for *in vitro *molecular analysis and the other for MR analysis. Following anesthesia, precise surgical methods were used to excise the GAS and SOL muscles from the hind limb. Muscles were then frozen in liquid nitrogen. Prior to removing the left calf muscles, a cardiac perfusion protocol was implemented to drain blood from the rat's body since it could interfere with the clarity of the imaging process.

### Diffusion tensor imaging (DTI) analysis for myofiber dimensions

**Figure 2:**
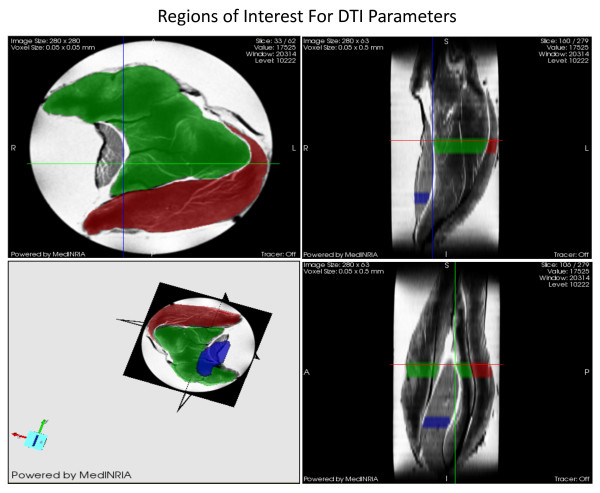
**Diffusion tensor imaging (DTI) of Rat Skeletal Muscle with Regions of Interest for the analysis**. Soleus muscle is marked with blue, while lateral and medial gastrocnemius muscles are marked with red and green, respectively.

For this study we were able to utilize the MR technique termed Diffusion Tensor Imaging (DTI) analysis to study muscle cell architecture at the NHMFL. DTI is based on the principle that the cellular diffusion of water corresponds to cell geometry in muscle. The advantage of DTI concerns the ability of random diffusion of water molecules to probe with far greater detail then general imaging techniques [26,27]. Unlike biopsy techniques, DTI is able to provide the average myofiber dimensions of an entire muscle, as opposed to a small sample of the muscle. Part of the DTI analysis involves calculating the mean diffusion of water within a muscle fiber (termed apparent diffusion coefficient, ADC), fractional anisotropy (FA) and the 3 principle directions of water diffusion denoted as Eigen vectors 1, 2 and 3, representative of the local fiber coordinate system [26,27]. The diffusive transport along the 3 principle directions are denoted as eigenvalues 1, 2, and 3 (λ1, λ2, and λ3) which correspond to diffusive transport along the long axis, as well as the long and short cross-sectional axes of the muscle fibers, respectively [28] (Figure 2). FA is a general measure of the differences in the magnitude of diffusion between the 3 principle directions of diffusion. With smaller cross sectional areas (CSA), FA increases while larger cross sectional areas decrease FA. Thus, FA is inversely proportional to myofiber size [26,27].

**Figure 3:**
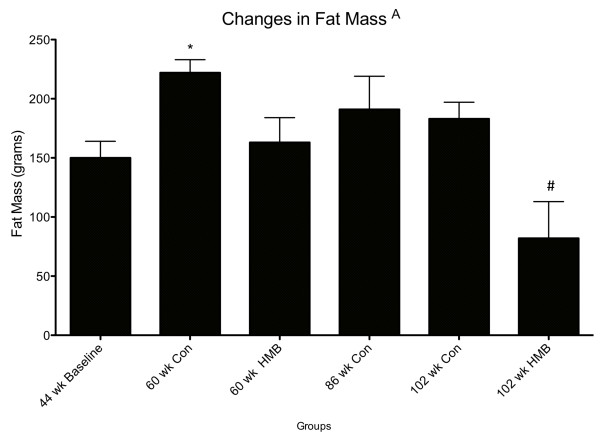
**Changes in fat mass among control and HMB conditions in young and older F344 rats**. Values are means ± standard deviations. A p < 0.05, main condition effect. * p < 0.05, significantly different from 44 wks baseline, $ significantly different from 86 wks baseline old.

DTI datasets of the muscles in 7-noncollinear gradient directions were acquired using a widebore 11.75-T vertical magnet with a Bruker Avance console and Micro2.5 gradients. Using a 15-mm birdcage coil, spin echo DTI scans were acquired with b values of 0, 500, and 1000 s/mm^2 ^at an in-plane resolution of 50 × 50 μm^2 ^and a slice thickness of 500 μm. The DTI acquisition parameters were as follows: TE = 20.5 ms, TR = 2.75 s, Δ = 12.7 ms and δ = 2.1 ms. Also, a high resolution (40-μm^3^) 3D gradient-recalled echo (GRE) image was acquired (TE/TR = 10/150 ms) for anatomical and volumetric measurements. After acquisition, the images were processed with MedINRIA http://wwwsop.inria.fr/asclepios/software/MedINRIA/ to calculate diffusion tensor parameters such as: FA, and λ1, λ2 and λ3. The region of interest (ROI) was chosen in the widest region of the GAS and SOL muscle for processing as shown in Figure 3.

### Semi-quantitative reverse transcription polymerase reaction (RT-PCR)

As previously described in detail we used a relative RT-PCR method using 18S ribosomal RNA as an internal standard was used to determine relative expression levels of target mRNAs [29]. We designed each set of forward and reverse primers using DNA Star Lasergene 7 software. All primer sets have been previously tested for optimal conditions. For each PCR reaction, 18S (with a 324-bp product) was co-amplified with each target cDNA (mRNA) to express each as a ratio of target mRNA/18S. Images were captured under UV, and mRNA expressions were analyzed via the Bio-Rad ChemiDoc™ XRS imaging system and the Bio-Rad QuantityOne^® ^software (Bio-Rad Laboratories, Hercules, CA, USA) as described previously [29].

mRNA expression of 4EBP1 was used as a negative marker of protein synthesis, while the E3 ligase atrogin-1 was used as a positive regulator of protein degradation. Mitogenic factors, IGF-IEa and its isoform IGF-IEb(mechano growth factor (MGF)), were used as positive regulators of mitogenesis and myogenesis. Myostatin and its receptor activin IIB were measured as negative regulators of myogenesis. Muscle cell regeneration was analyzed by transcriptional levels of the myogenic regulatory factors (MRFs): myogenin and myogenic differentiation factor (MyoD).

### Statistical analysis

Lean body mass, FM, TBM, functionality (grip strength and incline plane, MR-determined myofiber dimensions and target genes associated with myofiber size were analyzed using one way ANOVA across six groups including 1 young baseline (44 wks), 2 middle aged (60 wks, control and HMB), 1 old (86 wks.), and 2 very old (102 wks. control and HMB) groups using Statistica (StatSoft^®^, Tulsa, OK, USA) (Figure 1). Significance was set at p ≤ 0.05, and a tukey post hoc analysis was used to determine which specific mean values differed from others for each variable. The overarching goal of this project was to use MR to examine the impacts of age and HMB on skeletal muscle cells during the aging process. Myofiber size was therefore one of the primary outcome measures in this project and provided the basis for the sample sizes as determined by the G*Power analysis software [30,31]. Our rationale for sample size was based on a study by Payne et al. [32]. These investigators found that Fisher 344 rats 102 wks of age demonstrated significant atrophy in the soleus than young adult animals (Effect size (ES) of 3.7). Based on an alpha level of 0.05, a power of 80 and an ES of 3.7, a total of 30 rats (5 per experimental group) were needed to have sufficient power to detect age related changes in myofber dimensions.

## Results

### Food and HMB consumption

All values for food consumed are presented in Table 1. Average total Kcals and Kcals for carbohydrates, protein, and fat were not different between groups.

**Table 1:** Average Kcal consumption for among conditions

	Kcals	Kcals (CHO)	Kcals (PRO)	Kcals (Fat)
44 wks Baseline	67.3 ± 4.1	38.9 ± 2.4	19.2 ± 1.2	9.0 ± 0.6
60 wks Control	66.8 ± 1.8	38.7 ± 1.1	19.0 ± 0.5	8.9 ± 0.3
60 wks HMB	65.9 ± 1.5	38.2 ± 0.9	18.7 ± 1.2	8.8 ± 0.6
86 wks Baseline	62.3 ± 6.5	35.5 ± 3.64	17.4 ± 2.0	8.2 ± 0.9
102 wks Control	62.5 ± 5.8	36.1 ± 2.4	17.8 ± 1.0	8.4 ± 0.5
102 wks HMB	63.2 ± 6.19	36.8 ± 3.6	18.1 ± 1.8	8.5 ± 0.8

### Body composition

There were no condition effects for LBM. In regards to FM, there were significant condition (*p *≤ 0.05, ES = 0.5) effects, with greater FM (g) in the middle aged (60-wk) control (+49%) but not in the middle aged HMB condition, compared to the baseline young animals (Figure 3). Moreover, FM was significantly lower (-56%) in the very old HMB (102-wk) but not in the control condition compared to the 86 wk. old baseline animals.

### Functionality measures

All test reliability scores for functionality were above .9. There were significant condition (*p *≤ 0.05, ES = 0.7) effects for normalized grip strength in which strength was lower in the control condition, but was maintained in the HMB condition when comparing 44 to 60 wks. of age animals (Table 2). In old animals, normalized strength increased by 23% (p < 0.05) when comparing 86 to 102 wks. of age with HMB, with no change in the control condition. There was a condition effect (*p *≤ 0.05, ES = 0.4) for incline plane performance, which was greater in the 60 wk hmb condition than 44 wk condition, but was not different than baseline in the 60 wk control condition. Both old groups declined in incline plane performance relative to the 44 wk baseline group of animals.

**Table 2:** The Effects of Aging and HMB on Neuromuscular Function

	**Normalized Grip Strength**^**A**^	**Incline Plane (angle in degrees)**^**A**^
44 wks Control	4.5 ± 0.7	45.2 ± 1.7
60 wks Control	3.6 ± 0.3*$	47.6 ± 2.1
60 wks HMB	4.2 ± 0.4	51.0 ± 2.7*#
86 wks Control	3.3 ± 0.6*$@	40.0 ± 1.6*#$
102 wks Control	3.2 ± 0.6*$@	41.0 ± 1.6*#$
102 wks HMB	3.8 ± 0.5*	40.2 ± 1.7*#$

A indicates a main condition effect. * indicates p < 0.05, significantly different from 44 wks, $ indicates p < 0.05, significantly different from 60 wks HMB, # indicates p < 0.05, significantly different from 60 wks control, @ indicates p < 0.05, significantly different from 102 wks HMB

### Diffusion tensor imaging determined myofiber dimensions

**Figure 4:**
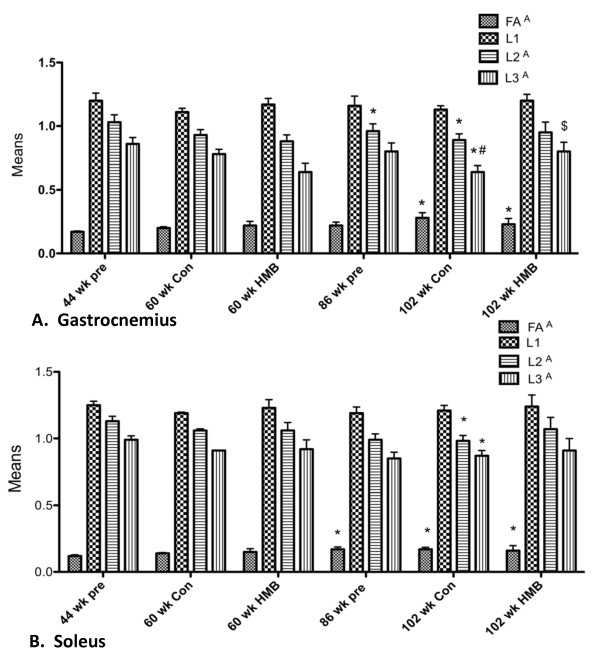
**Comparison of gastrocnemius and soleus muscle DTI data with or without HMB in young and older F344 rats**. A indicates a main condition effect (p < 0.05), * indicates a significant difference from the 44-wk group (p < 0.05), # p < 0.05, significantly different from 86 wk group, $ p < 0.05, significantly different from 102 wk HMB group.

We analyzed the GAS and SOL muscles and calculated the DTI parameters for those muscles (Figure 4). Fractional anisotropies (FA), apparent diffusion coefficients (AP), and eigenvalues [33] 1, 2, and 3 were investigated. There was a main condition effect for FA for the GAS (Figure 4A) (*p *≤ 0.05, ES = 0.5) and SOL (Figure 4B) (*p *≤ 0.05, ES = 0.5) muscles (Figure 4). Post hoc analysis revealed that while FA was significantly greater in the 102-wk control from both 44 and 86 wk., the 102-wk HMB condition only differed from 44 wk. No changes in FA occurred from 44 to 60 wk. in any of the conditions. There was a main condition effect for the GAS (*p *≤ 0.05, ES = 0.4) and SOL (*p *≤ 0.05, ES = 0.4) muscles for λ 2, indicative of myofiber CSA. There was also a main condition effect in the GAS (*p *≤ 0.05, ES = 0.4) and SOL (*p *≤ 0.05, ES = 0.4) muscles for λ 3, also indicative of myofiber CSA. Post hoc analysis revealed that λ 2 was lower (*p *≤ 0.05) in the SOL and GAS in the 86-wk and 102-wk control group. In addition λ 3 declined in the SOL of the 86-wk old condition, and in all muscle groups in the 102-wk control group. However, no changes occurred in the 102-wk HMB condition or any of the 60-wk conditions for any muscle analyzed. In the GAS, both λ 2 and 3 were greater in the 102-wk HMB than non-HMB condition. No condition effects were found for ADC, or λ 1, representative of diffusion in the longitudinal axis of the myofibers in any of the muscles analyzed.

### Semi-quantitative reverse transcription polymerase reaction

#### Regulators of protein turnover

**Figure 5:**
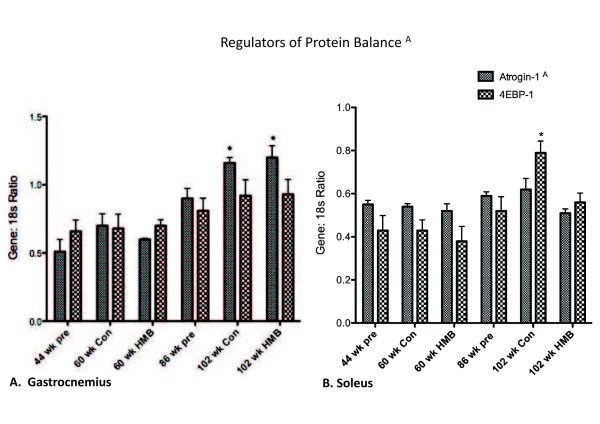
**Regulators of protein balance in the gastrocnemius and soleus muscles**. A indicates a main group effect (p < 0.05), * indicates a significant difference from the 44-wk group (p < 0.05).

No significant condition effects were found for either the SOL or GAS muscles for 4EBP-1 mRNA expression (Figure 5). However, there were significant condition effects for both the soleus (*p *≤ 0.05, ES = 0.5) and gastrocnemius muscles (*p *≤ 0.05, ES = 0.6) for atrogin-1 mRNA expression. There were condition effects for all muscles for atrogin-1, which was greater in the 102-wk control than all other groups in both the soleus (+ 45%) and gastrocnemius (+100%) muscles. However, the rise was blunted in the soleus in the 102-wk HMB condition.

#### Positive and negative regulators of mitogenesis

**Figure 6:**
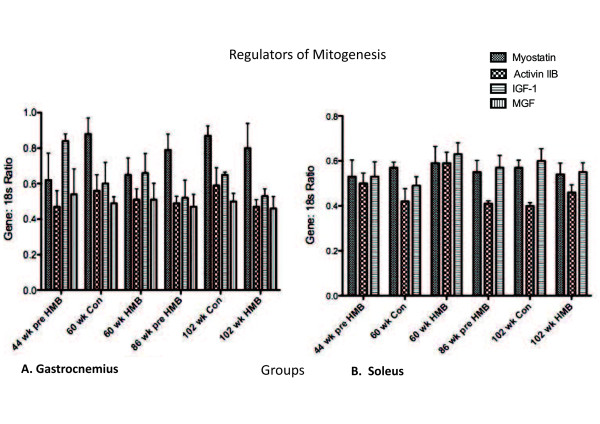
**Regulators of Mitogenesis in the gastrocnemius and soleus muscles**. * indicates a significant difference from the 44-wk group (p < 0.05).

Myostatin mRNA expression was too low in the soleus to process data. For the remaining data sets, no main effects were found for IGF-I, MGF, myostatin, or activin RIIB in any muscles analyzed (Figure 6).

#### Regulators of myogenesis

**Figure 7:**
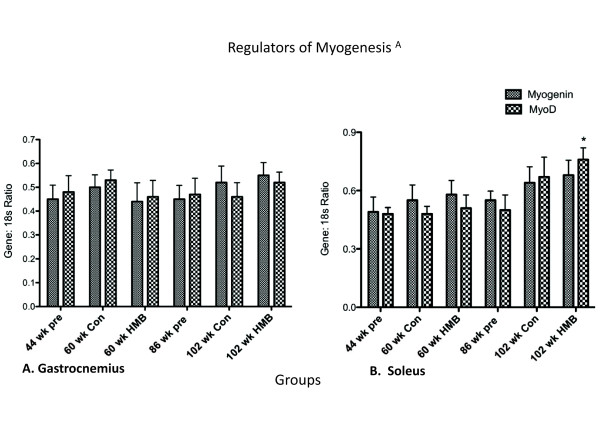
**Regulators of Myogenesis in the gastrocnemius and soleus muscles**. * indicates a significant difference from the 44-wk group (p < 0.05).

There were no main effects in the soleus or gastrocnemius for MyoD, or for the gastrocnemius in myogenin (Figure 7). However, there was a main group effect in the soleus for myogenin (*p *≤ 0.05, ES = 0.3) which while approaching significance in the 102-wk control group (p = 0.056) only significantly increased in the 102-wk HMB group relative to the 44-wk group.

## Discussion

The primary aim of the present study was to determine the effects of 16 wk. (approximately 15-16% of F344 rats normal lifespan) of HMB administration in young and old rats on age-related changes in body composition, myofiber dimensions, strength, and incline plane function. The major findings of this study were that HMB blunted negative age-related changes in body composition and muscle cellular dimensions.

### Body composition

**Figure 8:**
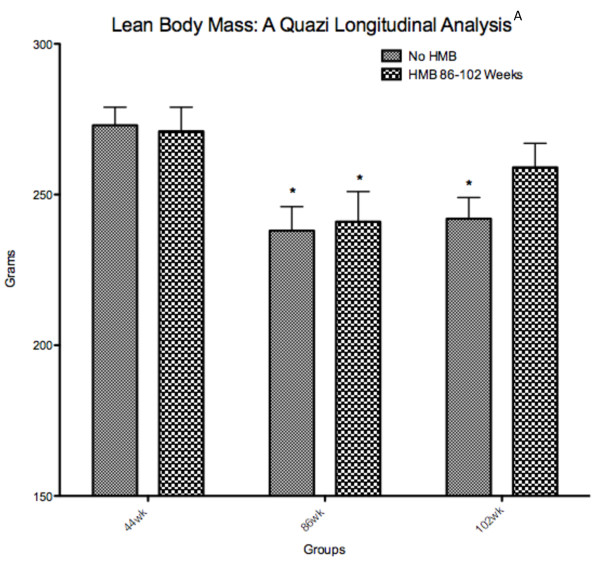
**Quazi longitudinal analysis of lean body mass in young (44 wk) to very old (102 wk)**. Fisher 344 rats. A indicates a main condition effect (p < 0.05), * indicates a significant difference from the 44-wk group (p < 0.05).

Results indicated no changes in LBM when comparing young to old rats. Our results agreed with Yu et al. [34] who also found that LBM did not change from young to old age in F344 rats. However, it is possible that the DXA measure of LBM in rats was not sensitive enough to detect age-related sarcopenia, and it's possible that the cross sectional design underestimates these changes. In general, both human and rodent models have shown to underestimate age-related changes in muscle mass when done in cross sectional designs relative to longitudinal designs [35-37]. Our old animals were raised in our laboratory from 44 to 86 weeks of age. While the HMB group continued (16-wk administration) until very old age (102 wk.), the control group was sacrificed at 86 wk. of age. Therefore, we performed a quazi-longitudinal comparison between the groups, in which a separate group of 5 control animals were used at 102 wk. in place of those 5 sacrificed at 86 wks. Intriguingly, both groups significantly declined in LBM from 44 to 86 wks. of age, and while this loss was maintained in the old control group, the 102-wk HMB group was no longer significantly lower in LBM than when they were 44 wk. of age (Figure 8). Baier et al. [38] also performed a longitudinal analysis in over 70 elderly women with an average age of 76 years of age. These subjects were randomly divided into either a cocktail containing HMB or placebo supplemented groups for a 12-month duration. Their results indicated that LBM progressively increased over a 12-month time span when supplementing with the nutrition cocktail with no change occurring in the placebo condition.

### Fat mass (FM)

In both humans and the Fisher 344 rat model, FM increases up to 70% of the lifespan, and then plateaus or decreases thereafter [39,40]. In our control rats, FM increased from young to middle age, with no changes occurring from old to very old age. Perhaps the most intriguing finding of our study was that HMB prevented fat gain from young to middle age, and significantly lowered body fat after the 16-wk HMB administration from the old to very old age. Our results also concur with past animal research, which demonstrated significantly lower hindlimb fat pad weight following HMB administration in both healthy and dystrophic mice [41]. Interestingly enough, these changes were independent of food intake, which agreed with past research indicating that grams of food consumed may not significantly change with age in the F344 rat model [42], nor with HMB supplementation. To date, the underlying mechanisms that HMB exerts its effects on adipose remain to be elucidated. It may be that HMB directly increases oxidative capacity in myofibers, as exposure of cultured myotubes to the leucine metabolite increased palmitate oxidation by 30% [43].

### Muscle strength and sensory motor function

The present study employed a direct measure of grip strength [24], as well as the incline plane test, which has been previously utilized to study both sensory motor function and whole body strength [25]. Sensory motor function is a combination of not only muscle strength, but motor unit recruitment and rate of muscle contraction [44]. For example, recovery of balance following sudden perturbations requires a quick and powerful reflex response to overtake the falling momentum [45]. There was an overall decline in grip strength from 44 to 102 wk. of age. When normalized to body mass however, grip strength declined from 44 to 60 wk. only in the control, but not in the HMB condition. Moreover, normalized grip strength increased by 23% in the old HMB condition from 86 to 102 wk. of age.

In addition, incline plane performance increased from young to middle aged rats that were administered HMB. Our results on overall functionality concur with Flakoll et al. [9] who previously demonstrated that 12 wk. of a cocktail containing HMB (also contained Arginine and Lysine) significantly increased grip strength, leg extension force, as well as get up-and-go performance in older adults. Finally, changes in functionality and strength without detectable changes in LBM may indicate an increase in muscle quality. However, this is currently speculative and would need to be verified by future research.

### Myofiber dimensions

Previous research with HMB supplementation has been restricted to indirect measures of muscle tissue which include caliper measurements [46,47], DXA analysis [38,48], and limb circumference measures [9]. However, the hallmark of sarcopenia is a decline in muscle mass and then ultimately in myofiber dimensions. To our knowledge, our study is unique as we are the first to view actual changes in muscle cellular dimensions following HMB administration throughout senescence. In particular, we employed the diffusion tensor imaging (DTI) technique, which uses a powerful magnet at the NHMFL. This technique has been validated for studying changes in myofiber dimensions including myofiber length and cross sectional area (CSA) following ischemia reperfusion injury [26,49,50]. As predicted, no changes occurred in myofiber dimensions from 44 to 60 wk. of age. While sarcopenia was evident in the 86-wk and 102-wk control conditions, both λ 2 and λ 3, indicative of myofiber CSA were relatively maintained in the soleus and gastrocnemius muscles of rats consuming HMB. Our results are consistent with previous work from Flakoll [9] and Bair et al. [38] who found that a cocktail containing HMB was able to counter age-related losses in limb circumference. These results are also consistent with several additional muscle wasting models which demonstrated HMB could blunt muscle loss during sepsis [51], cancer [16], limb immobilization [21], and in critically ill trauma patients [52].

### Transcript factors associated with myofiber size

Perhaps the most studied aspect of HMB is its effects on protein breakdown. The first research conducted was in humans, which demonstrated that HMB could significantly lower 3-methylhistadine following strenuous bouts of exercise [23]. However, only recently have its mechanisms of action been elucidated. The current study analyzed atrogin-1, an E3 ligase in the Ubiquitin pathway, which is commonly elevated in muscle wasting conditions such as aging [53,54]. We found that HMB was able to attenuate the age-related rise in atrogin-1 mRNA expression in the soleus muscle. This is important as atrogin-1 mRNA expression has been demonstrated to be a predictor of long-term changes in proteolysis and muscle wasting [55-57]. Moreover past research has found gene expression of atrogin-1 to be elevated in aging muscle tissue [55,56]. While our research analyzed HMB's effects on transcription of components of the Ubiquitin pathway, researchers in the Tisdale laboratory have studied direct activity of the Ubiquitin pathway [16,22]. These researchers found that HMB decreased proteasome activity, expression of both alpha and beta subunits of the 20s chamber, and the ATPase subunits of the 19 s caps.

Previous research from Baier and colleagues [38] found that whole body protein synthesis increased up to 14% during a 12-month period when subjects consumed an HMB containing cocktail. We looked at the effects of HMB directly in skeletal muscle on 4EBP-1 gene expression, the inhibitory binding protein that prevents formation of the eukaryotic initiation factor 2F complex which is rate limiting to translation initiation [58]. We did not see any aging or supplement effects on 4EBP-1. Our results agreed with Kovarik et al. [51] who found that HMB was able to attenuate a sepsis induced protein catabolic state in rat skeletal muscle primarily by blunting an increase in proteolysis, without preventing a decline in protein synthesis. However, a more recent study by Pimentel et al. [59] found that while HMB supplementation increased total mTOR protein expression, and phosphorylation of ribosomal protein s6 kinase (p70s6k) in healthy rats, that it was not able to increase the total protein expression of p70S6K. Thus the combined results from protein and gene changes from Pimental et al. [59] and our current study, respectively, may indicate that HMB does not directly regulate the expression of these two downstream targets of mTOR.

### Positive and negative regulators of mitogenesis and myogenesis

In our previous research with old female rats, we found that IGF-IEa mRNA expression was increased in a group administered HMB during 10-wk resistance training [60]. The current study found no significant main effects for myostatin, MGF, or IGF. However, past research found that the addition of HMB to serum-starved myoblasts increased IGF-I mRNA in a dose dependent manner. It is possible that the more robust effects seen in cell culture are due to a greater overall direct exposure of myocytes to HMB, as this study confirmed that HMB's effects on IGF-I were dose dependent. However, future research will need to be conducted to examine if higher doses elicit differential responses in animal studies.

MyoD and myogenin were taken as early and late regulators of satellite cell differentiation, respectively [61]. Our results showed a main group effect for myogenin in the soleus. However, this regulator of differentiation only significantly increased in the 102-wk. HMB condition, and not in the 102-wk. control condition. While it is tempting and certainly possible to suggest that HMB was at least partially responsible for this increase, it is more easily explained by a compensatory process accompanying the aging process [62] as the control condition very closely approximated a significant rise as well (p = 0.07).

## Conclusions

The prevalence of sarcopenia simultaneously increases along with the percentage of older individuals. It is often difficult to find an intervention that is adhered to by the elderly population than could possibly blunt this phenomenon. However, the results of our present study in sedentary rats indicate that HMB may prove efficacious in blunting deleterious changes in muscle mass and myofiber dimensions with age. Our findings of improved functionality with HMB also support previous findings observed in humans. Moreover, our findings demonstrate that HMB may have a catabolic effect on adipose tissue (fat mass), although underlying mechanisms in fat metabolism remain to be elucidated. While our study only began to elucidate the mechanisms this supplement works through, we did find that it lowered the E3 ligase atrogin-1, which is involved in a rate-limiting step in Ubiquitination of target substrates for degradation. It is suggested that future studies look directly at changes in myofiber growth with an in vivo MR DTI technique on the same animals over time concurrently analyzing changes in protein content of its regulators.

## Competing interests

The authors declare that they have no competing interests.

## Authors' contributions

J-SK was a PI for the present study responsible for funding, providing resources, study design, supervising data collection and tissue analysis, and manuscript preparation. JMW was responsible for study design, data collection, molecular and gene analysis, and manuscript preparation. SCG and IM assisted in study design, data collection and conducted the myofiber dimension analysis. S-rL, Y-mP and PCH assisted in data collection/analysis for the study, and harvesting of tissues. BHA and LBP assisted in funding, providing resources, and manuscript preparation. JRS and JPL helped extensively in manuscript preparation. All authors read and approved final manuscript.
